# Reinventing Guantanamo: From Detainee Facility to Center for Research on Neglected Diseases of Poverty in the Americas

**DOI:** 10.1371/journal.pntd.0000201

**Published:** 2008-02-27

**Authors:** Peter J. Hotez

**Affiliations:** Department of Microbiology, Immunology, and Tropical Medicine, George Washington University Medical Center and Sabin Vaccine Institute, Washington, D. C., United States of America

Reports that the Bush administration has expressed an interest in closing the Guantanamo Bay detainee facility in Cuba [1], colloquially known in the US as “Gitmo,” could stimulate a new chapter in US foreign policy. By converting Gitmo into a biomedical research institute dedicated to combating the diseases of poverty in the Western Hemisphere, we can tap into a little known, but highly effective tradition of vaccine diplomacy that we first began 50 years ago at the height of the Cold War [2],[3].

In the 1950s, polio epidemics were ravaging the major urban centers in the United States and in the Soviet Union. Children were the chief victims in both countries. The devastation wreaked by the polio outbreak spurred the establishment of new polio research laboratories in Moscow and in the US at Jonas Salk's laboratory in Pittsburgh, Pennsylvania and at Albert Sabin's lab in Cincinnati, Ohio.

Not long after the Sputnik launch in 1956, Washington put aside its ideological differences with the Soviets and permitted Mikhail Chumakov, the chief virologist at the Moscow laboratory, to collaborate with Dr. Sabin's laboratory to develop and test a live oral polio vaccine. Three years after Dr. Chumakov's historic visit, the Russians were vaccinating millions of children using an experimental vaccine developed in Dr. Sabin's lab. These initial Soviet trials provided the basis for the subsequent licensure of the oral polio vaccine throughout the US [2],[3]. As a result, almost every American inoculated against polio after 1963 and prior to 1996 received a vaccine that was developed and tested through Cold War diplomacy [2],[3].

Vaccine diplomacy could be similarly effective in reaching out to the poorest people in America's backyard.

In less than the time it takes to fly from Washington DC to the West Coast, I can walk through the public hospitals in Guatemala, Haiti, Honduras, and Nicaragua, and be guaranteed to see children suffering from protein malnutrition, parasitic worm infections, infectious diarrhea, Chagas disease, and tuberculosis meningitis. Data from the United Nations Children's Fund (UNICEF), World Health Organization, and the World Bank confirm this observation—some of the infant mortality rates and under-five child mortality rates in Bolivia, Guyana, and Haiti are as high as those in Botswana, Congo, Gabon, and Ghana [4]. The rates of malnutrition (moderate and severe stunting) in Guatemala, Haiti, and Honduras rival those in Chad, Somalia, and Zambia [5]. Indeed, five Latin American nations, namely Bolivia, Guyana, Haiti, Honduras, and Nicaragua, stand out for their low economic growth, low life expectancy, and high disease rates, presenting a bleak picture of poverty, malnutrition, and disease that compares with many regions of sub-Saharan Africa.

The poorest areas of Latin America and the Caribbean also stand out for their high rate of neglected tropical diseases, with millions or tens of millions of cases of hookworm and other intestinal worm infections [6], Chagas disease [7], schistosomiasis [8], and dengue [9], and almost one million cases of lymphatic filariasis and malaria [9] in the region. In a previous article published in *PLoS Neglected Tropical Diseases* it was pointed out that many of the neglected tropical diseases that now occur in Latin America and the Caribbean were first brought there during the Middle Passage of the Atlantic slave trade [10]. In this sense, the region's neglected tropical diseases represent a tragic living legacy of slavery [10].

America has an enormously impressive track record of developing and testing life-saving vaccines and drugs. It is therefore surprising that American leadership in biomedical sciences and its remarkable legacy for saving lives abroad has never been embraced as a meaningful component of American foreign policy. The US spends approximately $100 billion annually on health research and development, including $28 billion by the NIH and $57 billion by industry [11],[12].

The neglected tropical diseases represent some of the most important poverty-promoting disease conditions [13]. By establishing a center of excellence on the diseases of poor, at Guantanamo, the United States Government would directly address poverty and health disparities in the worst-off nations in Central and South America and the Caribbean. Such a center could conduct translational research to develop new drugs and vaccines for neglected diseases, possibly in collaboration with research institutes and public sector pharmaceutical manufacturers in some of Latin America's so-called *innovative developing countries*, such as Argentina, Brazil, Cuba, and Mexico [14]. It could also promote clinical research and take on the control of some of the more pressing public health threats in the Caribbean region, including vector-borne diseases such as dengue [9]. It would serve as a vital resource for training physicians, scientists, and public health experts, and meet an important demand for training in applying appropriate technology to global public health practice [15].

Through a focused initiative on lymphatic filariasis and schistosomiasis, a well-resourced biomedical institution located at Guantanamo could lead a path to eliminate these scourges from the Caribbean region [16] and forever wipe out an important element of slavery's legacy [10]. The facility could even take on some neglected tropical diseases that remain important yet often hidden public health problems among the economically disadvantaged and under-represented minorities living in the US, such as cysticercosis, dengue, leptospirosis, toxocariasis, and congenital cytomegalovirus infection and toxoplasmosis [17].

It is a moral outrage that a wealthy country like the United States allows its closest neighbors to suffer from some of the world's worst levels of disease, poverty, and malnutrition. Reinventing Gitmo to address our hemisphere's most pressing neglected health problems could help change America's reputation and legacy in the region. The US has previously supported covert military operations and encouraged military coups in the Americas [18], while the US Army continues to interrogate terrorist suspects at Gitmo (“We interrogate seven days a week, 24 hours a day,” a US general based at Gitmo told CBS news [19]). By transforming Gitmo from a detainee facility to a center for research on the diseases of poverty, the US would show that it sincerely wants to address the Millennium Development Goals in Latin America and the Caribbean [13], and ultimately make things better for the next generation of all Americans.

## Supporting Information

Alternative Language Abstract S1 Translation of the Article into Spanish by Maria Elena Bottazzi.
(0.05 MB DOC)
