# Supplementary material for: Reinventing Guantanamo: From Detainee Facility to Center for Research on Neglected Diseases of Poverty in the Americas
Source: PLoS Negl Trop Dis. 2008 Feb 27;2(2):e201. doi: 10.1371/journal.pntd.0000201 (PMC2254199; doi:10.1371/journal.pntd.0000201)
Supplement: Alternative Language Abstract S1 — Translation of the Article into Spanish by Maria Elena Bottazzi. (0.05 MB DOC) [file pntd.0000201.s001.doc]

# Reinvención de Guantánamo: De un Centro de Detención a un Instituto para la Investigación sobre las Enfermedades Desatendidas de la Pobreza en las Américas

Peter Hotez*

Departamento de Microbiología, Inmunología, y Medicina Tropical, George Washington University Medical Center y Sabin Vaccine Institute, Washington, D.C., Estados Unidos de America

**Conflicto de Intereses**: PJH es Director Ejecutivo de la Red Global para el Control de las Enfermedades Tropicales Desatendidas y Presidente del Sabin Vaccine Institute. És inventor de la EEUU Patente 7,303,752 B2 (publicada el 4 de diciembre del 2007) intitulada” Vacuna contra Uncinaria”

**Información del autor**: Peter J. Hotez es Editor-Jefe de *PLoS Neglected Tropical Diseases*, Profesor Walter G. Ross y Jefe de Departamento de Microbiología, Inmunología, y Medicina Tropical, y Presidente del Sabin Vaccine Institute.

Los informes donde la administración del Presidente Bush expresa un interés en el cierre del Centro de Detención de la Bahía de Guantánamo (Gitmo) en Cuba (1) podrían estimular un nuevo capítulo en la política exterior de los Estados Unidos. La conversión de Gitmo en un Instituto de Investigación Biomédica dedicado a combatir las enfermedades de la pobreza en el hemisferio occidental, podría explotar la poco entendida, pero altamente eficaz tradición de la vacunación diplomática que iniciamos hace 50 años en la cumbre de la Guerra Fría (2, 3).

En los años 50, las epidemias de poliomielitis estaban destruyendo los centros urbanos principales en los Estados Unidos y en la Unión Soviética. Los niños eran las principales víctimas en ambos países.  La devastación causada por los brotes de poliomielitis estimuló el establecimiento de nuevos laboratorios de investigación sobre la poliomielitis en Moscú y en Estados Unidos en el laboratorio de Jonas Salk en Pittsburgh, Pensilvania y en el laboratorio de Albert Sabin en Cincinnati, Ohio.

No mucho después del lanzamiento Spútnik en 1956, Washington puso a un lado sus diferencias ideológicas con los soviéticos y permitió a Mikhail Chumakov, el virólogo principal en el laboratorio de Moscú, de colaborar con el laboratorio del Dr. Sabin con objeto de desarrollar y evaluar una vacuna oral viva contra la poliomielitis. Tres años después de la visita histórica del Dr. Chumakov, los rusos ya vacunaban a millones de niños usando la vacuna experimental desarrollada en el Laboratorio del Dr. Sabin. Estos estudios soviéticos iniciales proporcionaron la base para la subsecuente licencia de la vacuna oral contra la poliomielitis a través de los Estados Unidos (2.3).  Como resultado, casi todo norteamericano inoculado contra la poliomielitis después de 1963 y antes de 1996 recibió una vacuna que fue desarrollada y evaluada a través de la vacunación diplomática de la Guerra Fría (2, 3).

La vacunación diplomática podría tener similar importancia para poder alcanzar y extender la ayuda a la gente más pobre que se encuentra en los países vecinos de América.

En menos del tiempo que se necesita para viajar de Washington DC a la costa oeste de Estados Unidos, yo puedo caminar a través de los hospitales públicos en Guatemala, Haití, Honduras, y Nicaragua, y tener certeza de poder ver a niños sufriendo de desnutrición proteínica, de infecciones por parásitos intestinales, de diarrea infecciosa, de enfermedad de Chagas, y de meningitis causada por tuberculosis. Los datos del Fondo para la Niñez de las Naciones Unidas (Unicef), de la Organización Mundial de la Salud, y del Banco Mundial confirman esta observación - algunas de las tasas de mortalidad infantil y tasas de mortalidad de niños menores de cinco años en Bolivia, Guyana y Haití son tan altas como las tasas observadas en Botswana, Congo, Gabón, y Ghana (4). Los índices de desnutrición (retardo de crecimiento moderado y severo) en Guatemala, Haití, y Honduras compiten con los índices encontrados en Chad, Somalia, y Zambia (5). De hecho cinco naciones Latinoamericanas, Bolivia, Guyana, Haití, Honduras, y Nicaragua, se destacan por su bajo desarrollo económico, años esperanza de vida, e índices de enfermedad, presentando un cuadro triste de la pobreza, desnutrición, y enfermedad que se compara con muchas regiones de Sub-Sahara África.

Las áreas más pobres de América Latina y el Caribe también se destacan por su alto índice de enfermedades tropicales desatendidas con millones o diez millones de casos de uncinaria y otras infecciones por parásitos intestinales (6), enfermedad de Chagas (7), schistosomiasis (8), y dengue (9), y casi un millón de casos de filariasis linfática y de malaria (9) en la región.  En un artículo publicado anteriormente en *PLoS Neglected Tropical Diseases* se menciona que muchas de las enfermedades tropicales desatendidas que ahora ocurren en América Latina y el Caribe fueron traídas originalmente durante el “paso del medio” del comercio de esclavos por el atlántico (10).  En este sentido, las enfermedades tropicales desatendidas de la región representan una real y trágica herencia de la esclavitud (10).

Ningún otro país puede igualar el record de sucesos que tiene Norte América para el desarrollo y evaluación de vacunas y drogas que salvan vidas. Por lo tanto es sorprendente que el liderazgo Americano en ciencias biomédicas y la notable herencia por salvar vidas en el exterior no ha sido nunca abarcado como un componente significativo de la política exterior americana. Estados Unidos gasta aproximadamente 100 mil millones de dólares anualmente en investigación y desarrollo para la salud, incluyendo 28 mil millones de dólares por el Instituto Nacional de Salud (NIH) y $57 mil millones de dólares por la industria (11, 12). Si esto fuese contado como parte de nuestra ayuda para el desarrollo exterior, los Estados Unidos empequeñecerían las contribuciones del grupo de ocho naciones combinadas.

Las enfermedades tropicales desatendidas representan una parte de las condiciones más importantes que promueven las enfermedades de la pobreza (13). Por medio del establecimiento de un centro de excelencia en Guantánamo dirigido al estudio de las enfermedades de los pobres, el gobierno de Estados Unidos trataría directamente con los problemas de la pobreza y las disparidades de salud en naciones de Centro y Suramérica y el Caribe con peor estado económico. Dicho centro podría conducir investigación aplicada para el desarrollo de nuevas drogas y vacunas contra las enfermedades desatendidas, posiblemente en colaboración con institutos de investigación y productores farmacéuticos dentro del sector público y ubicados en países de Latinoamérica conocidos como *países innovadores en vías de desarrollo*, como por ejemplo Argentina, Brasil, Cuba, y México (14). Podría también promover la investigación clínica y adquirir el control de algunas de las amenazas más acuciantes de salud pública en la región del Caribe, incluyendo enfermedades transmitidas por vectores tales como dengue (9). Serviría también como un recurso vital para la preparación y enseñanza de médicos, científicos, y expertos en salud pública, y crear la importante demanda para el entendimiento de la aplicación de tecnología apropiada hacia la práctica de salud pública global (15).

Mediante una iniciativa enfocada en filariasis linfática y schistosomiasis, una institución biomédica con altos recursos y situada en Guantánamo podría conducir una trayectoria para la eliminación de estas enfermedades de la región del Caribe (16) y eliminar para siempre un elemento importante derivado de la herencia de la esclavitud (10). La institución incluso podría tomar algunas de las enfermedades tropicales desatendidas que siguen siendo de importancia, aunque a menudo problemas ocultos de salud pública, entre la población de bajos recursos y las minorías que viven en los Estados Unidos, y que consisten en enfermedades tales como cisticercosis, dengue, leptospirosis, toxocariasis, infección congénita por citomegalovirus y toxoplasmosis (17).

Es un insulto moral que un país rico como los Estados Unidos permite que sus vecinos más cercanos sufran de algunos de los peores niveles de enfermedad, pobreza, y desnutrición del mundo. La reinvención de Gitmo para combatir en nuestro hemisferio los problemas desatendidos de salud mas urgentes podría ayudar a cambiar nuestra reputación y herencia en la región, de una reputación que anteriormente apoyaba operaciones militares secretas y alentaba los golpes militares (18), y que aun hoy interroga a sospechosos terroristas, a una reputación que desea sinceramente lograr las Metas de Desarrollo del Milenio en Latino América y el Caribe (13), y en última instancia crear cosas mejores para la generación futura de todos los americanos.

Bibliografía

1. BBC News. Rice in Guantanamo closure appeal.  <http://news.bbc.co.uk/go/pr/fr/-/2/hi/americas/7155142> , accessed December 29, 2007.
2. Benison S. International medical cooperation: Dr. Albert Sabin, live poliovirus vaccine and the Soviets.  *Bull Hist Med*  1982; 56: 460-83.
3. Hotez PJ. Vaccines as instruments of foreign policy.  *EMBO Reports*  2001; 2: 862-8.
4. <http://www.unicef.org/sowc05/english/Table1_E.xls>, accessed December 29, 2007.
5. <http://www.unicef.org/sowc05/english/Table2_E.xls>, accessed December 29, 2007.
6. De Silva NR, Brooker S, Hotez PJ, Montresor A, Engels D, Savioli L.  Soil-transmitted helminth infections: updating the global picture. *Trends Parasitol*  2003; 19: 547-51.
7. Franco-Paredes C, Von A, Hidron A, Rodriguez-Morales AJ, Tllez I, Barragan M, Jones D, Naquira CG, Mendez J.  Chagas disease: an impediment in achieving the Millennium Development Goals in Latin America.  *BMC Int Health Human Rights*  2007; 7:7.
8. Chitsulo L, Engels D, Montresor A, Savioli L. The global status of schistosomiasis and its control.  *Acta Tropica*  2000; 77: 41-51.
9. Pan American Health Organization. *Health in the Americas 2007*, Volume I – Regional, Scientific and Technical Publication 622.
10. Lammie PJ, Lindo JF, Secor WE, Vasquez J, Ault SK, Eberhard ML. Eliminating lymphatic filariasis, onchocerciasis, and schistosomiasis from the Americas: breaking a historical legacy of slavery.  *PLoS Negl Trop Dis*  2007; 1: e71.
11. <http://www.futurepundit.com/archives/003004.html>, accessed December 29, 2007.
12. Zerhouni EA. US biomedical research.  *JAMA*  2005; 294: 1352-8.
13. Hotez PJ, Molyneux DH, Fenwick A, Kumaresan J, Ehrlich Sachs S, Sachs JD, Savioli L. Control of neglected tropical diseases.  *N Engl J Med*  2007; 357: 1018-27.
14. Morel CM, Acharya T, Broun D, Dangi A, Elias C, Ganguly NK, Gardner CA, Gupta RK, Haycock J, Heher AD, Hotez PJ, Kettler HE, Keusch GT, Krattiger AF, Kreutz FT, Lall S, Lee K, Mahoney R, Martinez-Palomo A, Mashelkar RA, Matlin SA< Mzimba M, Oehler J, Ridley RG, Senanayake P, Singer P, Yun M.  Health innovation networks to help developing countries address neglected diseases.  *Science*  2005; 309: 401-4.
15. Hotez PJ, Gupta R, Mahoney R, Poste G. Incorporating appropriate technology into North American schools of public heatlh. *Pan Am J Publ Health*  2006; 19: 118-23.
16. Rawlins SC, Lammie P, Tiwari T, Pons P, Chadee DD, Oostburg BFJ, Baboolal S. Lymphatic filariasis in the Caribbean region: the opportunity for its elimination and certification.  *Pan Am J Public Health*  2000; 75: 319-24.
17. Hotez PJ. Neglected diseases and poverty in “The Other America”: the greatest health disparity in the United States?  *PLoS Negl Trop Dis*  2007; 1: e149.
18. Gil L. *The School of the Americas: Military Training and Political Violence in the Americas*, Duke University Press 2004; 296pp.
